# Effect of COVID-19 restrictions and fuel prices on traffic volume and offenses in Iran: A spatiotemporal analysis

**DOI:** 10.1371/journal.pone.0332443

**Published:** 2025-10-23

**Authors:** Milad Delavary, Amir Hossein Kalantari, Hossein Farsangi, Abolfazl Mohammadzadeh Moghaddam, Ali Hadianfar, Ward Vanlaar, Martin Lavallière

**Affiliations:** 1 Department of Health Sciences, Laboratoire BioNR and Centre intersectoriel en santé durable (CISD), Université du Québec à Chicoutimi, Chicoutimi, Québec, Canada; 2 Traffic Injury Research Foundation, Ottawa, Ontario, Canada; 3 Safety and Security Science Section, Department of Values, Technology and Innovation, Faculty of Technology, Policy and Management, Delft University of Technology, Delft, Netherlands; 4 Department of Civil Engineering, Sharif University of Technology, Tehran, Iran; 5 Department of Civil Engineering, Faculty of Engineering, Ferdowsi University of Mashhad, Razavi Khorasan, Iran; 6 Noncommunicable Diseases Research Center, Neyshabur University of Medical Sciences, Neyshabur, Iran; University of Valencia, SPAIN

## Abstract

With the outbreak of the COVID-19 pandemic and the subsequent imposition of mobility restrictions in many nations, traffic volumes and driving behaviors have changed worldwide. This study aims to investigate the effect of COVID-19 restrictions and fuel prices on traffic volume and offenses (speeding, tailgating, and illegal overtaking) in Iran’s provincial and aggregated data in the study period of March 21, 2019, to May 20, 2020. A time-series analysis was conducted to capture the effects of interventions in level and trend, followed by a spatial autocorrelation of the interventions among provinces to identify the provinces that formed clusters in terms of traffic volume and offenses before and after each intervention. Most of the COVID-19 restrictions (and the pandemic itself) did not reduce traffic volume and rate of traffic offenses whereas an increase in fuel prices decreased traffic volume and offenses (except for illegal overtaking). Furthermore, traffic volume showed an increasing trend after the imposition of mobility restrictions, suggesting that preventive measures could not control intercity trips during the pandemic. Spatiotemporal analysis showed mobility restrictions effectively removed some provinces from the clusters with above-average volume, tailgating, and overtaking data. The possible reasons for these findings and potential solutions are discussed.

## Introduction

Motor vehicle collisions (MVCs) are responsible for 1.35 million deaths each year. Ninety-three percent of these deaths occur in low- and middle-income countries, which own approximately 60% of the world’s vehicles [1]. Various factors can contribute to MVCs; however, risky driving behaviors, such as speeding [2–5], tailgating [6,7] and illegal overtaking [8,9] are some of the most prominent traffic offenses that could lead to a collision. In Iran, 17,803 road users died in 2019 [10], and from March 2019 to March 2020, 276,771 people were injured in MVCs [11]. Moreover, according to the findings of the Global Burden of Disease 2010, MVCs, with 5%−10% of all deaths, were one of the leading causes of death in Iran [12].

In December 2019, the COVID-19 virus (SARS-CoV-2) was detected in Wuhan, China [13], and on March 11, 2020, a pandemic was declared by the World Health Organization. Lockdowns were imposed in many countries, activities were limited to only the most essential ones, and people were prohibited from leaving their residential premises [14]. Consequently, important restrictions on mobility were applied, which prohibited national and international roads and air travel [15–17]. This resulted in a rapid decline in traffic volume on the roads of several countries [18–21] and changes in driving behaviors, such as speeding with most of the studies showing increasing trends [22–25]. These changes, in turn, caused substantial changes in MVCs worldwide, with most of the countries showing a downward trend [26–28]. This is in line with previous studies stating that the probability of MVCs decreases with a decrease in the volume of vehicles on roads [29]. On February 19, 2020, the first two confirmed cases of COVID-19 death in Iran were recorded [30] and the day after, the Iranian government announced the closure of schools and other educational institutes until March 2 and 5, respectively [31]. Additionally, on March 27, 2020, just a week after starting the new Persian year (Nowruz, a holiday that spans approximately two weeks), the first nationwide mandate was announced, which restricted any national travel on roads and public gatherings, even in open spaces such as parks. As part of this mandate, individuals were urged to observe “social distancing” regulations [32]. These global and national findings have encouraged researchers to investigate how pandemic-induced mobility restrictions and behavioural shifts affected road safety outcomes—particularly traffic volume, driving behaviours, and motor vehicle collisions (MVCs). While such studies have yielded valuable insights, particularly in countries with robust traffic surveillance systems, questions remain regarding the extent to which these findings can be generalised—especially to low- and middle-income countries such as Iran, where policy implementation, enforcement, and mobility patterns may differ considerably.

Despite the growing body of research examining the effects of COVID-19 on traffic patterns and safety, findings remain inconsistent—largely due to differences in data sources, analysis methods, and geographic scope. Part of the existing literature relies on descriptive statistics, offering limited insight into temporal or spatial trends. Moreover, many studies are confined to specific cities or regions, making it difficult to generalize findings at a national level. In the context of Iran, relevant research remains scarce and is often limited to self-reported data or single-province case studies, highlighting the need for a more comprehensive and methodologically robust national analysis.

In light of the above context, this study aims to address the following objectives:

- Conducting an interrupted time series (ITS) analysis on traffic volume and offenses (as safety indicators) and comparing these indicators in three stages of pre, during, and post restriction periods.
- Conducting a spatiotemporal analysis assessing spatial autocorrelation between neighboring provinces, with the aim of identifying which regions were most affected by the COVID-19 restrictions before and after each intervention.
- Investigating the ITS analysis findings for the provincial data on a case-by-case basis and see if the results align with the aggregate data (the whole country’s data), which this part is available in the online supplementary material.

The remainder of this paper is structured as follows. First, previous studies regarding the effect of COVID-19 on traffic safety and driving behaviors are reviewed. Next, the methodology is described. Subsequently, we present and discuss the results of the study. Finally, conclusions are drawn based on the results obtained.

### Literature review

#### Effect of the COVID-19 pandemic on traffic volume, collisions, and behaviors.

As mentioned, the COVID-19 restrictions in most countries resulted in notable changes in road traffic volume, MVCs, and traffic behaviors. Overall, previous studies mostly compared the first few months of the pandemic when the movement restrictions were in place to the same period of the first year of the pandemic (2019) and found a reduction from 9 to 93 percent in traffic volume compared to the pre-lockdown period [18,25,33–36]. Some studies also showed the negative effect of the COVID restrictions on specific types of trips such as work-related trips [19]. Moreover, research has shown that even in some countries such as Japan [37] with no strong governmental restrictions and penalties regarding daily mobility, a significant reduction in inter-and intra-city trips was observed, which also did not return to the normal level just after the lifting of these restrictions.

Furthermore, studies regarding MVCs show when considering the crash data as an aggregate, a reduction from 38 to 60 percent was evident in some European and Asian countries [27,34,38,39] with the caveat that the extent of the reduction in MVCs during the pandemic was greater than the decrease in traffic flow in some cases [28]. Also, road deaths declined from 10 to 64 percent [19,27,34,39]. Regarding the USA, data from Florida, New York, and Massachusetts suggested an average reduction of 494.39, 62.06, and 332.97 total vehicle collisions per day, respectively [40]. Also, the data from five other states in the U.S. showed that the pandemic was associated with a 20% reduction in vehicular crashes and an estimated savings of up to $16 billion if travel restrictions were in place until the end of June 2020 [41].

However, some studies suggest that when the collision data were stratified, an increase in the number of crashes and crash intensity was observed [29,42]. For example, in the US, it was reported that during the COVID, rates for injury, non-injury, and fatal crashes involving single vehicles increased by 1.76, 2.55, and 2.29 times, respectively, compared with the past three years and only crash rates for all crash types involving multiple vehicles reduced significantly [29]. It was also suggested although both slight and serious injuries were reduced in Northern Ireland, approximately the same number of people died as would have been anticipated in the absence of the pandemic [42]. The lack of change in the number of deaths could be attributed to the increased spatial extent and higher vehicular speeds on roads resulting from the reduced traffic volume, as reported in many studies [23,24,42–44].

Studies regarding speeding found an increase in average speeding from 2 to 39 percent [36,38]. Liao and Lowry (2021) studied speeding-related collisions in New York City and Seattle and observed that fatal and severe injury crash rates of speeding-related MVCs increased 4.5 times in New York City and 7.7 times in Seattle [22]. Tucker and Marsh (2021) stated that several factors could explain speeding and aggressive driving during the pandemic, including a reduced optical edge rate and motion parallax magnitude, absence of time-to-collision information, lower visual demand, perceived risk of the driving task, and increased boredom on roads as a matter of low traffic volume [23].

While earlier research on the effects of COVID-19 on mobility and motor vehicle collisions (MVCs) in Iran was limited, a growing body of studies has since emerged. For example, Nadimi et al. (2022) examined the impact of the pandemic on both rural and urban transportation in Iran, highlighting reduced demand for public transport, changes in trip purposes, and an increase in driving speed [45]. Similarly, Kolivand et al. (2024) conducted an interrupted time series analysis using national data and found a significant decline in road traffic injuries and fatalities during the early months of the pandemic, followed by a gradual increase in the subsequent months [46]. A survey regarding mobility habits and perceived risks of using transportation systems before and during the travel restrictions in 10 countries including Iran showed that people reported a 22.1% increase in “no-use” of all transport modes. Also, Iran had the highest rate of change in response to “never walk” during the restrictions compared to the pre-restrictions period suggesting more people refrained from going outside during this period [47]. A study in Tabriz, East Azerbaijan showed that COVID restrictions significantly decreased the number of urban MVCs in the early stage of the pandemic compared to the previous year [48].

Overall, as evident from the existing literature, discrepancies among findings are apparent, primarily attributed to variations in data collection methods and the types of data utilized. Many studies have relied solely on descriptive analyses, which, while valuable, offer a limited depth of understanding regarding the patterns of changes in traffic safety indicators (such as the number/rate of MVCs and traffic offenses). Additionally, a majority of these studies have focused on a restricted number of cities or states, potentially limiting their ability to represent the broader national context. Furthermore, studies pertaining to Iran are notably scarce, often limited to self-report studies or single-case analyses, typically confined to a single city or province.

Therefore, this study aims to investigate the impact of the first wave of COVID-19 restrictions on Iran’s provincial data related to traffic volume and offenses over a 14-month period, spanning from March 21, 2019, to May 20, 2020. To gain a more comprehensive understanding of how these measures may have influenced traffic behaviors and safety in Iran, we employed a spatiotemporal analysis. Additionally, we included the rise in fuel prices as a control intervention, allowing us to compare its effects with those of the COVID-19 restrictions.

Previous research has shown that when fuel prices increase, similar to the pandemic, road users decrease their mileage and drive more cautiously [49,50], the pattern of non-essential trips change, and traffic volume decreases [51]. Moreover, the rate of collisions and fatalities resulting from other driving behaviors, such as speeding and braking change [52,53], and the number of related MVCs declines [49,52,54–56].

Informed by this body of literature and contextualized within Iran’s dual exposure to pandemic-related restrictions and fuel price reforms, the following methodological approach was designed to evaluate the temporal and spatial impact of these interventions on traffic volume and violations across provinces.

## Methodology

### Data preparation and description

For this study, data for road traffic offenses (RTO) and traffic volumes were obtained from the Iranian Road Ministry website, which publishes statistics for daily RTO in the 31 provinces of Iran [57]. The aggregated datasets are provided in Supporting Information (S1 File for RTO and S2 File for traffic volumes).These data are publicly available only for intercity (rural) roads, which are maintained by the Iranian Road Ministry’s jurisdiction. However, urban roads are maintained by municipalities, where such data are recorded but not published; therefore, they were not available for this study. The daily provincial RTO and volume data for 14 months (427 days from March 21, 2019, to May 20, 2020) were gathered and the incidence rates of speeding, tailgating, and illegal overtaking per 1000 vehicles were considered for the analyses. The RTO data were recorded by traffic counter sensors (i.e., inductive loops) which were installed on intercity (rural) roads. The inductive loops measure changes in the electric field when objects pass over them. Traffic volume in model outputs was divided by 10,000,000 for simplicity. Speeding is detected when the subject vehicle exceeds the road’s speed limit. The speed limits on main roads are 95 km/h during the day and 85 km/h at night. On side roads, the limits are 85 km/h during the day and 75 km/h at night. Freeways have a speed limit of 120 km/h for passenger cars and 110 km/h for other vehicles. Moreover, illegal overtaking is detected when a vehicle crosses a solid line marking (i.e., a no-overtaking line) on the road in a direction opposite to that of the installed inductive loops. For example, if the loop is installed from left to right on the road, and the detected vehicle movement is in the opposite direction. Finally, tailgating is defined based on the speed limit of each road section and the minimum headway (time gap) of two seconds. This is calculated as the time when the rear axle of the lead vehicle is detected to the time when the front axle of the following vehicle is recorded [57]. The daily number of observed offenses per 1,000 vehicles (the incidence rate) was considered for the analysis.

Table 1 presents a list of important road safety events in Iran during the study period which was the first wave of COVID restrictions. It is worth mentioning the minimum time lag between the two interventions in our data was 10 points (days). This was between the start of the pandemic and school closure showing that Iran was faster in closing schools after the first confirmed cases than the world average [58]. This is in line with the literature stating that there should be at least 6 and 7 points to investigate subsequent interventions and achieve a reliable model [24,59]. Detailed province-level results of the interrupted time-series (ITS) analyses, including supplementary tables are provided in Supporting Information S3 File.

**Table 1:** Key transportation and policy events in Iran during the study period.

Event	Date of occurrence	Description
The rise in Fuel prices	11/15/2019	Each driver with a fuel car would have to pay 15,000 rials (13 US cents) per liter for the first 60 liters of petrol bought each month and 30,000 rials (50% increase) for each additional liter after 60 liters of petrol bought each month [60]. This is the only fuel price increase in the studied period. Note that in Iran, unlike many countries, the fuel prices do not change daily and there were few instances where the price changed in recent years including the previous rise from 7,000–10,000 rials in July 2014 [61] and the implementations of such acts are nationwide and simultaneously for all the provinces in Iran. Reducing energy subsidies is a potential approach to alleviate fiscal strain, as it can increase government revenue, a measure commonly taken after a recession [62], and is also implemented as a road safety measure.
Start of the Pandemic	02/19/2020	The first two confirmed cases of COVID-19 death in Iran were recorded [30]; this was announced in national news.
School Closure	02/29/2020	Iran’s Ministry of Health declared the closure of schools and higher educational institutions nationwide to curb the spread of the COVID-19 virus [63].
Imposing Moving Restrictions	03/27/2020	The first restriction was announced restricting any national travel on roads and public gathering even in open spaces such as parks; individuals were urged to observe “social distancing” regulations [32].
Removing Restrictions	04/19/2020	The nationwide restriction was lifted.

### Statistical analysis

#### Time-series analysis.

In this study, to capture the effects of the aforementioned interventions (Table 1) on traffic volume and offenses, a dynamic regression model with a seasonal autoregressive integrated moving average (SARIMA) residual was used with the following format [64]:


$$y=\beta_{\text{0}}+\beta_{t}T+\sum_{i=\text{1}}^{n}\beta_{li}L_{i}+\beta_{ti}T_{i}+N_{t}$$ (1)

where:


$$N_{t}\sim ARIMA(p,d,q)(P,D,Q)$$

T = Number of days elapsed since the start day of data.

n = 5; 1 to 5 included increasing fuel prices, the start of the pandemic, school closure, and imposing and removing movement restrictions respectively.

*Β*_*0*_ = Intercept of the model.

*Β*_*t *_ = Trend of elapsed time.

*Β*_*ti *_ = Amount that trend changes as a result of intervention i.

*Β*_*li *_ = Amount that level changes as a result of each intervention.

$L_{i}$ *=* Dummy variable, zero outside of i’s intervention period, and one otherwise.

$T_{i}$ = Number of days elapsed after i’s intervention during, otherwise zero.

The first part of the model is a piecewise linear regression model that captures intervention effects in level and trend, and the second part is a SARIMA model that captures the remaining patterns in residuals.

Autocorrelation function (ACF) and partial autocorrelation function (PACF) plots were used to know the order of autoregressive (AR) and moving average (MA) models. In addition, the stationarity was checked with these plots and Dicky-Fuller (DF) test, and it was confirmed for all provinces as well as the aggregate data of Iran. Furthermore, testing for white noise was done using *wntestq* in Stata to see whether the variables followed a white noise process. We also checked for the normality of residuals with the Kolmogorov Smirnov (KS) test [64]. Checking for the short-and long-term nonlinearity was done using Teraesvirta and White Neural Network tests (The null is the hypothesis of linearity in “mean”) [65,66] and decay functions such as log function were tested and indicated that the slope does not take the log form. The results of these tests are available by request. All of the analyses were conducted on all 108 models (26 [provinces] + 1 [Iran] for four variables). In order to account for chance capitalization and balance type I & II errors, p < 0.01 was considered as the significance level based on Sidak’s adjustment formula for multiple testing [67]. Finally, to check whether residuals had a normal distribution with a mean of zero, the KS test was used.

### Spatial autocorrelation

As COVID-19 is an infectious disease with high contagiousness, the spread of the virus in a specific geographical area can have a direct relationship with the neighboring regions as this could make these regions implement similar restricting policies and plans to control the spread. This could, in turn, affect traffic volume and behavior. Hence, investigating the autocorrelation between these regions (different provinces of Iran) could be helpful to understand some of the results of the temporal analysis better. Spatial autocorrelation represents the correlation between the values of similar variables concerning different locations and with this assumption that a relationship exists between different values of a variable regarding their distance and orientation relative to each other [68]. Anselin in 1995 [69] proposed Local Moran’s I, which can be used to measure the similarity and dissimilarity of each location pair from adjacent geographical areas. The local Moran’s I statistics of spatial autocorrelation is defined as


$$I_{i}=\frac{x_{i}-\overset{―}{x}}{s_{i}^{\text{2}}}\sum_{j=\text{1},i\neq j}^{n}w_{i,j}(x_{i}-\overset{―}{x})$$ (2)

where $s_{i}^{\text{2}}$ represents variance; $x_{i}\;$ and $\bar{x}$ are the value and mean of the variable for region i, respectively and $w_{i,j}$ is the spatial weight between region i and j.

A positive test value (p < .05) shows geographical areas have similarities for high-high and low-low clusters. A high-high cluster, for example, means that a region has a significantly high value, and its neighbors have high values as well (the first “high” refers to the studied region, and the second “high” refers to its neighbors). If the test result is negative (p < .05), it suggests an outlier. For instance, a high-low outlier means that a region has a significantly high value while its neighbors have low values. A Low-high outlier means that a region has a significantly low value while its neighbors have high values. A p-value higher than 0.05 shows no spatial autocorrelation [70]. In this study, p = 0.05 was considered as the significant level for the spatial analysis as we conducted one test per response variable.

All the descriptive maps and spatial analyses in this study were created by the authors using ArcGIS software, version 10.2.

## Results

**Fig 1:**
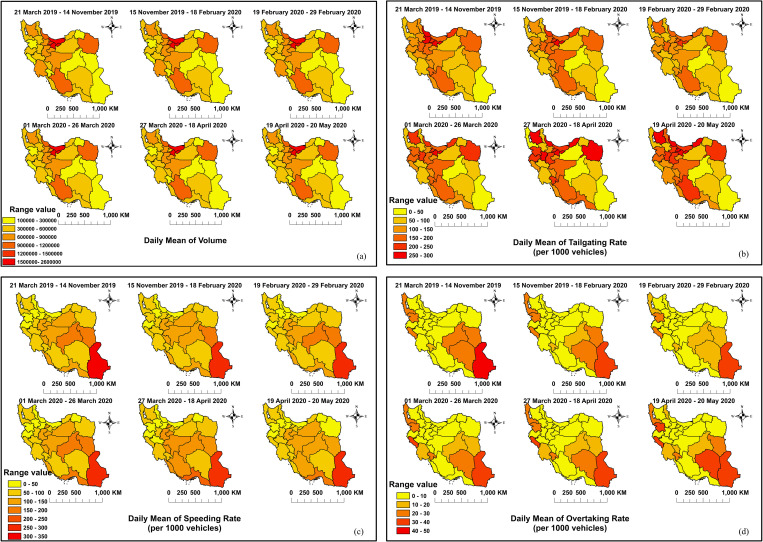
Data Description of (a) traffic volume and offenses including (b) speeding, (c) tailgating and (d) illegal overtaking. The figure was created by authors using ArcGIS software version 10.2.

Fig 1 shows spatiotemporal maps of traffic volume and offenses, including illegal overtaking, speeding, and tailgating, which were obtained by averaging the daily rates of each province before and after each intervention. Five provinces, Hormozgan, Qazvin, West Azerbaijan, Hamedan, and Khuzestan, were excluded from the models owing to missing information. However, they were included in the maps, which show the mean daily rate of the examined variables because the means could be calculated despite the missing data. Some provinces, in their offense data, had anomalies that were detected with an anomaly detection function (the underlying algorithm is referred to as seasonal hybrid extreme studentized deviate), which builds upon the generalized extreme studentized deviate test [71]. In the other provinces, where the number of missing data was insignificant, missing data were interpolated with the “na” interpolation function (in this function, missing values are imputed by values of approx, spline, or stinterp interpolation [71].

From Fig 1a, it can be seen that the daily traffic volume was highest in the north and lowest in the southeast during the study period. Fig 1b shows the rate of the daily mean of tailgating changed substantially during this period. For instance, Fars in the south, East Azerbaijan in the northwest, and Razavi Khorasan in the northeast showed an increase in average daily RTO during the study period, especially after the movement restrictions. As shown in Fig 1c, speeding was higher in southeastern provinces (e.g., Sistan and Baluchestan) and lowest in northern Iran (e.g., Mazandaran). Additionally, the mean daily rate of illegal overtaking (Fig 1d) in the southeastern (e.g., Sistan and Baluchestan) and northwestern (e.g., Western Azerbaijan) provinces was the highest during the study period.

**Fig 2:**
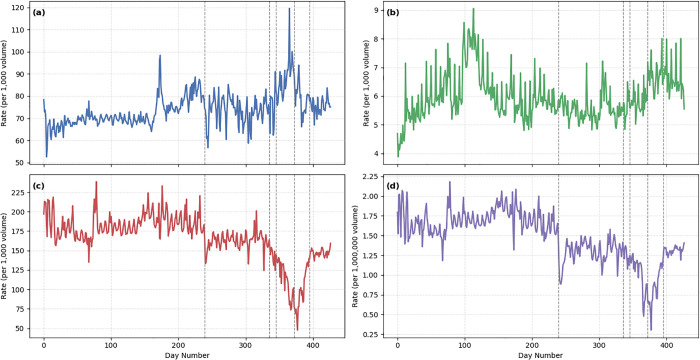
Trends in daily mean traffic violations and volume across the study period (March 21, 2019 – May 20, 2020). Subplots (a) to (d) show, respectively, the rate of speeding, illegal overtaking, tailgating (all per 1,000 traffic volume), and overall traffic volume (per 1,000,000) across all provinces. Dashed vertical lines indicate five major interventions: fuel price increase, pandemic onset, school closures, movement restrictions, and easing of restrictions.

Fig 2 presents the temporal trends of key traffic indicators, including rates of speeding, overtaking, tailgating (per 1,000 volume), and total traffic volume (per 1,000,000), over the 14-month period. The first major intervention—the increase in fuel prices—coincides with a sharp drop in traffic volume (subplot d) and a moderate yet visible decline in speeding and overtaking rates (subplots a and b), suggesting a reduction in overall travel activity and possibly more cautious driving behavior due to higher costs. However, the tailgating rate (subplot c) remained relatively stable during this period, indicating that certain risky behaviors may be less price-sensitive or more context-dependent.

Subsequent interventions associated with the COVID-19 pandemic had more pronounced and sustained effects. The onset of the pandemic and school closures were followed by increased volatility in offense rates, particularly for tailgating. The implementation of movement restrictions corresponds with a sharp reduction in traffic volume and a noticeable drop in offense rates across all categories, likely reflecting both reduced mobility and heightened public compliance. Finally, the removal of restrictions was associated with a gradual return in traffic volume and an uptick in some offenses, although the rates did not fully rebound to pre-pandemic levels within the study period. Together, these patterns underscore the responsiveness of traffic behaviors to both economic and public health interventions, with varying sensitivity across different types of offenses.

### ITS results in Iran

Table 2 shows the comparison of baseline models to ITS models based on Akaike information criterion (AIC). As evident from the table, all ITS models showed a lower AIC compared with their bassline counterparts suggesting better performance.

**Table 2:** Comparing the baseline models with ITS models.

Time series	Models	AIC^1^
	Interrupted time series; error: SARIMA(1,0,2)(0,1,1)_7_	−670.30
Volume	Baseline Model 1: SARIMA(1,0,2)(0,1,1)_7_	−650.47
	Baseline Model 2: SARIMA(1,0,1)(0,1,1)_7_	−634.64
	Interrupted time series; error: SARIMA(1,0,0)(0,1,1)_7_	2377.68
Speeding	Baseline Model 1: SARIMA(3,0,0) (4,1,0)_7_	2439.90
	Baseline Model 2: SARIMA(3,0,0) (0,1,1)_7_	2399.74
	Interrupted time series; error: SARIMA(1,0,0)(0,1,1,7)_7_	3160.87
Tailgating	Baseline Model 1: SARIMA(1,0,2)(0,1,1)_7_	3192.53
	Baseline Model 2: SARIMA(2,0,1)(0,1,1)_7_	3193.85
	Interrupted time series; error: SARIMA(2,0,0) (0,1,1)_7_	534.15
Illegal Overtaking	Baseline Model 1: SARIMA(2,0,0)(2,1,0)_7_	558.15
	Baseline Model 2: SARIMA(0,1,1)(2,1,0)_7_	572.83

1 Akaike information criterion.

Table 3 shows the results of the ITS analysis of traffic volume and rate of traffic offenses in Iran during the study period. As shown in Table 3, the increase in fuel prices caused a significant reduction in the level of traffic volume and the rates of speeding and tailgating (p < .001) but did not show any significant change in the trend of these variables as well as the level and trend of illegal overtaking. Moreover, the start of the pandemic and school closure did not exhibit any significant changes in the four studied variables.

**Table 3:** Results of ITS analysis for traffic offenses and volume in a total rural road in Iran.

	Volume (1/10^7)				Speeding (per 1,000 vehicles)				Tailgating (per 1,000 vehicles)				Illegal overtaking (per 1,000 vehicles)			
Type of Intervention	Estimate	P	CI (95%)		Estimate	P	CI (95%)		Estimate	P	CI (95%)		Estimate	P	CI (95%)	
			L	U							L	U			L	U
**InL1** ^**1**^	**−0.37**	**<0.01**	**−0.47**	**−0.27**	**−7.10**	**<0.01**	**−11.96**	**−2.23**	**−26.80**	**<0.01**	**−39.72**	**−13.87**	**−0.11**	**0.76**	**−0.86**	**0.63**
**InL2**	**0.03**	**0.85**	**−0.33**	**0.40**	**−2.57**	**0.49**	**−9.90**	**4.75**	**−3.31**	**0.83**	**−34.59**	**27.95**	**−0.06**	**0.92**	**−1.48**	**1.34**
**InL3**	**0.15**	**0.44**	**−0.24**	**0.56**	**−1.31**	**0.78**	**−10.82**	**8.19**	**22.42**	**0.07**	**−2.35**	**47.20**	**−0.01**	**0.98**	**−1.85**	**1.82**
**InL4**	**−0.14**	**0.27**	**−0.39**	**0.11**	**−12.45**	**<0.01**	**−21.13**	**−3.77**	**−18.88**	**0.06**	**−38.90**	**1.14**	**0.79**	**0.20**	**−0.44**	**2.02**
**InL5**	**1.05**	**<0.01**	**0.32**	**1.78**	**−38.00**	**<0.01**	**−54.34**	**−21.66**	**120.23**	**<0.01**	**63.41**	**177.06**	**0.91**	**0.52**	**−1.91**	**3.74**
**InT1** ^**1**^	**0.05**	**0.80**	**−0.41**	**0.52**	**−10.05**	**0.19**	**−25.23**	**5.12**	**3.14**	**0.84**	**−28.16**	**34.46**	**−1.00**	**0.63**	**−5.19**	**3.18**
**InT2**	**−2.06**	**0.36**	**−6.47**	**2.35**	**81.80**	**0.08**	**−9.63**	**173.23**	**−351.24**	**0.06**	**−718.28**	**15.80**	**3.35**	**0.59**	**−9.04**	**15.75**
**InT3**	**0.05**	**0.97**	**−4.18**	**4.30**	**−18.45**	**0.67**	**−105.89**	**68.97**	**109.90**	**0.54**	**−241.20**	**461.02**	**−2.53**	**0.68**	**−14.66**	**9.60**
**InT4**	**1.04**	**<0.01**	**0.39**	**1.68**	**−25.57**	**<0.01**	**−39.20**	**−11.93**	**128.00**	**<0.01**	**80.72**	**175.28**	**0.22**	**0.84**	**−2.02**	**2.47**
**InT5**	**0.75**	**0.09**	**−0.13**	**1.65**	**−19.18**	**0.13**	**−44.33**	**5.97**	**82.59**	**0.02**	**10.75**	**154.43**	**−0.78**	**0.61**	**−3.85**	**2.29**
**Noise**	**SARIMA(1,0,2)(0,1,1)**_**7**_				**SARIMA(1,0,0)(0,1,1)**_**7**_				**SARIMA(1,0,0)(0,1,1,7)**_**7**_				**SARIMA(2,0,0) (0,1,1)**_**7**_			
**Wntestq** ^**2**^	**0.71**				**0.45**				**0.25**				**0.95**			
**KS** ^**3**^	**0.11**				**0.91**				**0.99**				**0.61**			

1. InL1 to InL5 and InT1 to InT5 represent the five interventions in the level and trend, respectively: the rise in fuel prices, the start of the pandemic, school closure, imposing movement restrictions, and removing the restrictions.

2. White noise Q test.

3. Kolmogorov Smirnov test.

Meanwhile, mobility restrictions did not change the volume immediately, but a significant increase was observed in the long term (trend) (p < .001). This intervention could reduce speeding in the level and trend (p < .001). Also, tailgating showed a significant increase in the trend (p < .001) but no change in the level. However, illegal overtaking did not indicate any significant changes among the variables.

Finally, the removal of the restrictions significantly increased traffic volume and tailgating in the level (p < .001) but showed no effect on the trend of these variables. Moreover, speeding showed a significant drop in the level (p < .001) but no effect on the trend. Also, this intervention caused no significant changes in illegal overtaking.

### Spatial autocorrelation of the interventions among provinces

**Fig 3:**
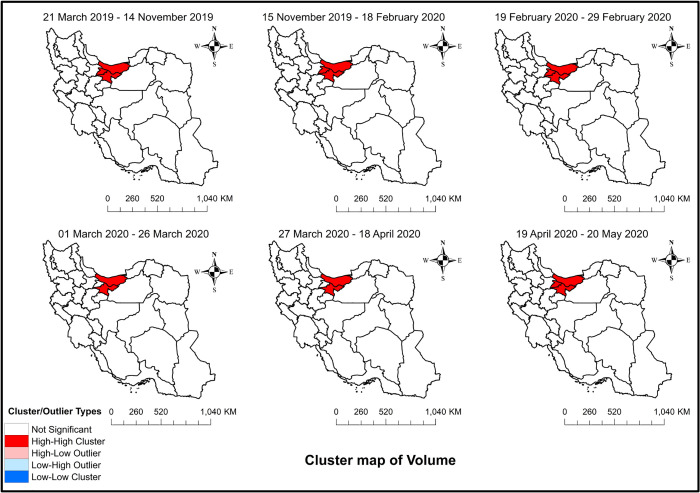
Spatial autocorrelation (Moran’s I) cluster maps of traffic volume among the different provinces of Iran: From March 21, 2019 to May 20, 2020 – Study period. The figure was created by authors using ArcGIS software version 10.2.

Fig 3 shows a map of the spatial autocorrelation of traffic volume (Moran’s I) among different provinces of Iran. According to Fig 3, Mazandaran, Alborz, and Tehran formed a high-high cluster from the start of the study period till when school closure was announced, meaning that these regions had above-average volume and they also shared boundaries with neighboring regions that had above-average values of this variable in the mentioned period. However, after the school closure, Alborz was removed from the cluster, and this continued until mobility restrictions were uplifted, where the initial cluster was formed again.

**Fig 4:**
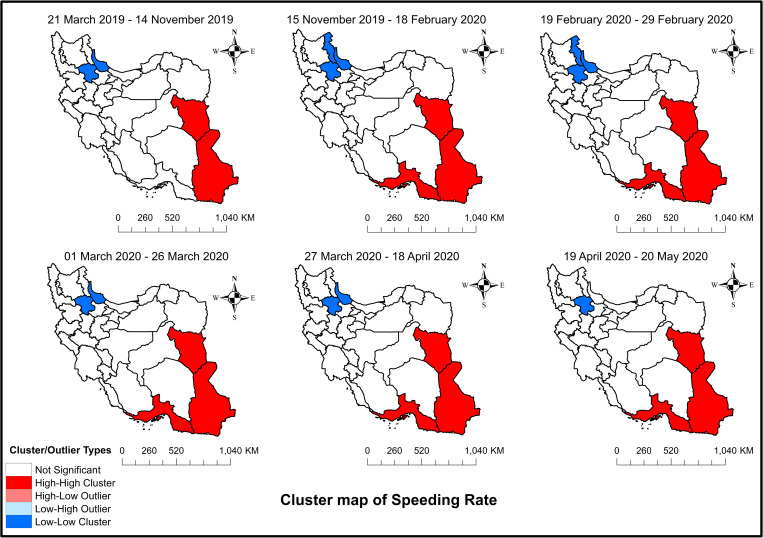
Spatial autocorrelation (Moran’s I) clusters maps of speeding among the different provinces of Iran: From March 21, 2019, to May 20, 2020 – Study period. The figure was created by authors using ArcGIS software version 10.2.

Fig 4 shows maps of Moran’s I of speeding among the different provinces of Iran during the study period. Before the increase in fuel prices (upper-left panel), Sistan, Baluchestan, and South Khorasan formed a high-high cluster (highlighted in red). Conversely, Gilan and Zanjan formed a low-low cluster (highlighted in blue), meaning these regions had below-average rates of speeding, and they also shared boundaries with neighboring regions that had below-average values of this variable. After the rise in fuel prices, East Azerbaijan and Hormozgan were added to the low-low and high-high clusters, respectively. This clustering can also be seen after the start of the pandemic; however, East Azerbaijan was no longer a part of the low-low cluster after the school closure and imposition of restrictions. Although the high-high cluster remained unchanged between the mentioned provinces until the end of the study period, the low-low cluster was observed only in Zanjan after removing the restrictions, suggesting that only this province had a significantly lower average speeding rate.

**Fig 5:**
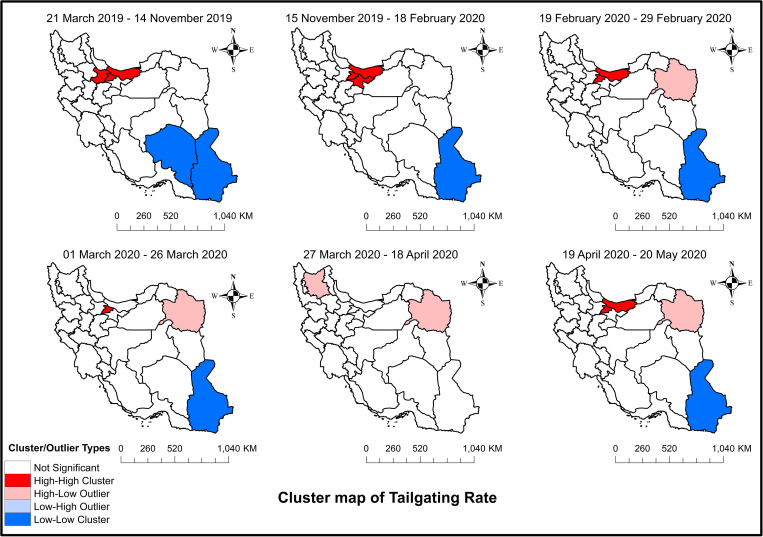
Spatial autocorrelation (Moran’s I) clusters maps of tailgating among the different provinces of Iran: From March 21, 2019, to May 20, 2020 – Study period. The figure was created by authors using ArcGIS software version 10.2.

Fig 5 shows maps of Moran’s I for tailgating among the different provinces of Iran during the study period. As shown in Fig 5, before the increase in fuel prices, Mazandaran, Qazvin, and Alborz formed a high-high cluster, whereas Sistan, Baluchestan, and Kerman formed a low-low cluster. After the first intervention, Qazvin was substituted with Tehran in the high-high cluster and Kerman was removed from the low-low cluster. After the start of the pandemic, Tehran was no longer a part of the high-high cluster, whereas Razavi Khorasan was in the high-low outlier, meaning the risk of occurrence of illegal overtaking was significantly high in this province and low in the surrounding provinces. School closure kept Alborz in the high-high cluster, with no change in the other clusters. The imposition of restrictions resulted in two high-low outliers, namely Razavi Khorasan and East Azerbaijan. Finally, removing the restrictions caused the observed high-high cluster between Mazandaran and Alborz and the low-low cluster between Sistan and Baluchestan to resurface.

**Fig 6:**
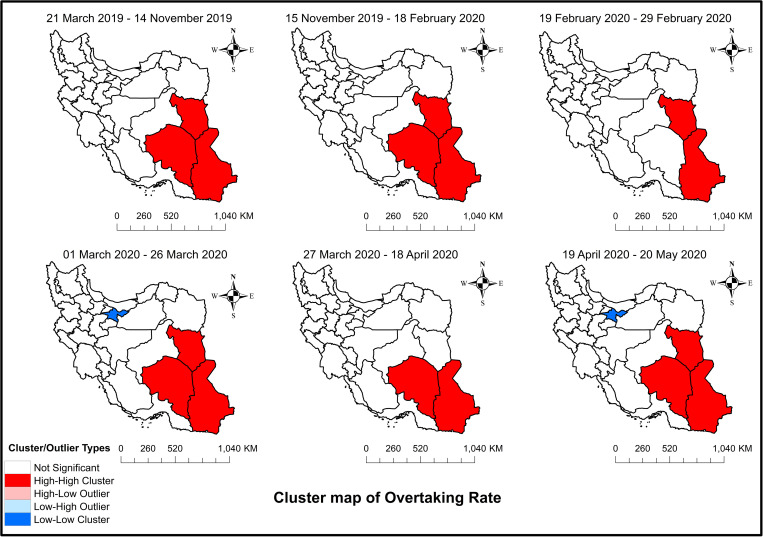
Spatial autocorrelation (Moran’s I) clusters maps of illegal overtaking among the different provinces of Iran: From March 21, 2019 to May 20, 2020 – Study period. The figure was created by authors using ArcGIS software version 10.2.

Fig 6 shows maps of Moran’s I for illegal overtaking among the different provinces of Iran during the study period. As can be observed, Sistan, Baluchestan, South Khorasan, and Kerman were in a high-high cluster before the pandemic. Kerman was removed from the cluster after the pandemic. According to the map for school closure, Tehran and its neighboring provinces formed a low-low cluster, and Sistan, Baluchestan, South Khorasan, and Kerman formed a high-high cluster. Finally, the imposition of the restrictions only resulted in a high-high cluster for Kerman, Sistan, and Baluchestan, and removing them led to clusters similar to the pre-restriction period.

## Discussion

In this study, we investigated the effect of five interventions on traffic volume and three types of traffic offenses over a 14-month period (i.e., the first wave of COVID-19 restrictions) in Iran using spatiotemporal analysis. The main contributions of our study were the analysis of the data of Iran, considering the effects of COVID-related interventions separately and comparing it to our control intervention (fuel prices), and then using spatiotemporal analysis to identify those provinces that formed a cluster regarding RTO and volume data. Our study distinguishes itself by examining the combined effects of COVID-19 restrictions and the concurrent rise in fuel prices on traffic safety metrics, offering a more nuanced understanding of the factors influencing driver behavior during this period. While previous studies have often focused on specific regions or relied on aggregated national data, our spatiotemporal analysis provides a detailed assessment of provincial-level variations over a 14-month period. The findings reveal that the impact of interventions varied significantly across different areas. These discrepancies may be attributed to differences in study design, including the level of geographic granularity, the duration of the analysis period, and the inclusion of overlapping interventions such as fuel price fluctuations. By employing high-resolution daily data and advanced statistical modeling, our study may have captured effects that were overlooked or diluted in more aggregated analyses.

The results of the temporal analysis showed that traffic volume decreased after the increase in fuel prices, remained unchanged after the start of the pandemic and school closure, showed an unexpected rise in the trend after the imposition of restrictions, and finally showed an increase in the level after these restrictions were removed. Although a significant number of provinces had a reduction in traffic volume after the rise in fuel prices (21 out of 26, S3–S1 Tables), the Moran’s I showed they were not correlated to each other, and the decline happened individually without each province affecting the neighboring provinces. Moreover, the results of Moran’s I showed that both school closure and mobility restrictions could remove a province from a high-high cluster of traffic volume, suggesting the limited effect of these interventions. Thus, while school closures had a regional effect, they did not lead to any significant change in interprovincial travel among Iranians. This suggests that the increase in traffic volume after the start of the pandemic was not due to the leisure time provided as a result of school and educational institute closures. School closure can play a role in people’s (especially students and pupils) usage of different transport modes and has been found to negatively affect the traffic volume of urban roads [72] although this aspect was not the focus of our study. Future studies should investigate the effect of COVID-19 restrictions on urban roads in Iran as well.

Overall, while the results after the rise in fuel prices were expected, the start of the pandemic and the imposition of mobility restrictions did not reduce the traffic volume in Iran. This suggests people did not substantially change their travel behaviors during the pandemic and the preventive measures were not sufficiently effective to reduce inter-city trips. A possible reason could be the high-high cluster of the two provinces (including the capital) with high populations, which remained unchanged after the restrictions (Fig 3). Previously, an origin-destination analysis of inter-provincial trips in Iran showed that these two provinces maintained a high flow of population movement even during the pandemic [73]. The increase in volume after removing restrictions corresponds with longer-term studies such as Kim and Kwan’s study in the USA [74] observing that people’s mobility levels decreased in the early stages of COVID but soon recovered to pre-pandemic levels and remained at similar levels, although there was a more severe second surge of newly confirmed COVID-19 cases.

When considering the results of speeding in Iran, it is observed that the increase in fuel prices led to a reduction in the level of speeding. However, the trend showed no significant change, suggesting that the effects were long-lived. This aligns with previous studies indicating that higher gasoline prices can lead to reduced speeds, as drivers adjust their speeds to consume less fuel per distance driven [53,75]. However, neither the start of the pandemic nor school closure showed any effect on Iran’s speeding data. From the data perspective and based on the provincial data (S3−S2 Tables), only a handful of provinces showed an increase after the pandemic and a reduction after the school closure. This probably explains why there was no significant effect on the aggregated data. Nevertheless, the imposition of mobility restrictions could reduce speeding in the level and trend. Unlike most other countries, the traffic volume in Iran did not change in the level and even indicated an increase in the trend after restrictions. This could explain why speeding also showed contradictory results to what was expected as previous research showed there is a negative association between traffic volume and speeding [23,24,42]. This can also be confirmed by the speed-density relationship in traffic science, where traffic density (a measure of the number of vehicles on roads in an area) has a linear relationship with the speed with a negative slope; hence, with an increase in density, the speed of the roadway decreases [76]. Some countries such as Australia showed similar results where a sample of Australian drivers reported reduced speeding during the restrictions [77]. Finally, removing restrictions could reduce speeding in the level that can again be confirmed by observing the volume data.

Next, the rate of tailgating decreased after the increase in fuel prices concerning the level but remained unchanged with respect to the trend, which can be confirmed by observing the provincial data (S3 Tables). The start of the pandemic and school closure did not influence tailgating at either the level or trend. These results corresponded to the observed volume and speeding data. However, according to Moran’s I results, the start of the pandemic could remove a province from a high-high cluster of three provinces, and this continued by removing another province after the school closure and removing the cluster completely after mobility restrictions. The movement restrictions did not cause a sudden change in tailgating, but the trend increased significantly with 13 provinces showing an increasing trend (S3–S4 Tables). Tailgating has a direct relationship with traffic volume and is more serious during rush hours [78]. The relatively stable traffic volume after this intervention might suggest that the restrictions did not drastically reduce the number of vehicles on the road, potentially leading to concentrated periods of higher traffic density and tailgating, especially during essential travel times. Also, drivers might have maintained closer distances between vehicles, possibly leading to an increase in tailgating incidents as drivers adjusted their following distances in slower-moving traffic (Table 3). Eventually, as expected, removing the restrictions resulted in an increase in tailgating both in the level and the trend, which also matches the findings of the volume data [78].

Finally, data on illegal overtaking for Iran showed no change because of the interventions. This is elucidated in the provincial data. For each intervention, there were two groups of provinces (approximately the same number) that could neutralize each other’s effects, whereas some provinces remained unchanged. Nevertheless, Moran’s I result revealed that mobility restrictions effectively removed a province from a high-high cluster, and the removed province returned once the restrictions were lifted.

## Conclusion

This study analyzed nationwide traffic data to investigate the effect of COVID-19 restrictions and the increase in fuel costs on traffic safety metrics in Iran. Overall, the results showed that an increase in fuel cost reduced traffic volume and speeding and tailgating instances on intercity roads in Iran. Moreover, the imposition of mobility restrictions caused an increase in traffic volume, which showed that preventive measures were unsuccessful in controlling intercity trips during the pandemic. This could explain, among other reasons such as a low rate of vaccination among the population [79], why Iran experienced consecutive peaks of the disease since the start of the pandemic. Initiatives should be implemented to minimize intercity travel during pandemics in order to mitigate the transmission of current or future infectious diseases. This can be achieved by adding and installing new smart cameras and sensors at the exit points of cities, as well as implementing fines specifically targeting intercity travel. Moreover, spatial analysis could help identify those provinces that formed a cluster for each variable. Temporarily increasing fines and attaching negative points to driving licenses could be another method of controlling mobility. This was observed to be effective among Iranian drivers [61] and could be a short-term solution. Additionally, while the restrictions could decrease speeding, they caused tailgating to increase in the long run, which could be related to the possibility of how crowded the roads were during the study period or an indicator of how drivers’ behaviors might have changed. Finally, the removal of restrictions led to an increase in traffic volume and tailgating, alongside a decrease in speeding, suggesting that—consistent with findings from previous studies—both speeding and tailgating are influenced by traffic density and overall driving conditions. Accordingly, systematically examining and comparing the effects of various interventions on daily traffic volume and the incidence of traffic offences throughout the pandemic can provide valuable insights for policymakers and traffic planners. Such analyses enable a clearer understanding of how specific measures impact traffic safety indicators, thereby informing more effective policy responses in future public health crises.

This study has several limitations that should be acknowledged. First, five provinces were excluded from the spatiotemporal analysis due to missing or unreliable data, which may limit the generalizability of the findings. Second, due to the macroscopic scale of the analysis, regional differences in driving behavior could not be fully explored, and the relationship between traffic volume and traffic offenses was modeled as linear for simplicity. Third, the dataset lacked demographic information such as drivers’ age, sex, and driving experience, which restricted deeper analysis of behavioral patterns underlying the observed trends. While these limitations do not undermine the primary contributions of this study—namely, the use of a national dataset and robust analytical methods—they highlight important avenues for future research aimed at enhancing the granularity and interpretability of traffic safety analyses in Iran.

Future research can build on the findings of this study by exploring several key avenues. One potential direction involves investigating the effects of COVID-19-related interventions on urban roadways and comparing them with intercity road settings, where traffic patterns and driver behavior may differ significantly. Additionally, examining how various socio-demographic groups (e.g., age, gender, income) respond to traffic interventions and offenses could yield insights for designing targeted and equitable safety strategies. To support such analyses, relevant data sources should be identified in alignment with existing RTO data or through a mixed-methods approach involving surveys and interviews. This would also facilitate an exploration of the psychological and behavioral factors driving increased traffic offenses during mobility restrictions. Furthermore, longitudinal studies are needed with longer periods of study to assess whether pandemic-induced changes in driving behavior are temporary or indicative of lasting shifts. Longer data periods would also help address the imbalance between pre- and during-COVID-19 timeframes and improve control over potential seasonal biases. Due to data constraints, we were also unable to determine whether the observed impacts were short-lived or indicative of long-term behavioural or policy shifts. Moreover, evaluating the effectiveness of technological interventions and law enforcement strategies across different phases of the pandemic could further inform best practices for future crisis management. Lastly, investigating the impact of increased telecommuting and remote work on traffic volume and safety could offer valuable insights for long-term transportation planning and policy.

## Supporting information

S1 File Aggregated data-traffic offenses. (XLSX)

S2 File Aggregated data-traffic volumes. (XLSX)

S3 File Supplementary Material for Effect of COVID-19 Restrictions and Fuel Prices on Traffic Volume and Offenses in Iran: A Spatiotemporal Analysis. (DOCX)
