# Supplementary material for: Effect of COVID-19 restrictions and fuel prices on traffic volume and offenses in Iran: A spatiotemporal analysis
Source: PLoS One. 2025 Oct 23;20(10):e0332443. doi: 10.1371/journal.pone.0332443 (PMC12548865; doi:10.1371/journal.pone.0332443)
Supplement: S3 File — (DOCX) [file pone.0332443.s003.docx]

Supplementary Material for Effect of COVID-19 Restrictions and Fuel Prices on Traffic Volume and Offenses in Iran: A Spatiotemporal Analysis

Milad Delavary, Amir Hossein Kalantari, Hossein Farsangi, Abolfazl Mohammadzadeh Moghaddam, Ali Hadianfar, Ward Vanlaar and Martin Lavallière

**Effect of interventions on different provinces of Iran**

Tables S1 to S4 show the results of the ITS analysis of traffic volume and the rate of traffic offenses in Iran during the study period. The rows of the tables show the number of provinces that were affected significantly either by one of four types of interventions that the ITS models can capture (i.e. both level and trend showed a significant change) or in the level/trend only. Based on the coefficients estimated by the model, four types of interventions can be captured by the model: upward shift with an increasing trend (Type 1), upward shift with a decreasing trend (Type 2), downward shift with an increasing trend (Type 3), and downward shift with a decreasing trend (Type 4). Table S1 shows that the increase in fuel prices caused 21 out of 26 provinces to decline significantly in the level of traffic volume and one province showed a drop in the level of this variable with an increasing trend. Moreover, Table S2 indicates 12 out of 26 provinces showed a reduction in speeding with respect to either the level/trend or both. This number was 16 out of 26 for tailgating as shown in Table S3. Additionally, three provinces showed an increasing trend after this intervention. Results regarding Table S4 and illegal overtaking showed a decline in the level or trend of this variable in five provinces while four provinces also showed a rise in the level or trend.

The start of the pandemic caused no significant change in both the level and trend of traffic volume, tailgating, and illegal overtaking in all provinces. Four provinces, however, showed an increasing trend in speeding.

**Table S1. Results of ITS analysis for traffic volume in provincial data of Iran**

Intervention

| Observed pattern | Fuel prices | Start of the pandemic | School closure | Imposing restrictions | Removing restrictions |
| --- | --- | --- | --- | --- | --- |
| Only Upward Shift | 0 | 0 | 0 | 0 | 9 |
| Only Downward Shift | 21 | 0 | 0 | 0 | 0 |
| Only Increasing Trend | 0 | 0 | 0 | 19 | 0 |
| Only Decreasing Trend | 0 | 0 | 0 | 0 | 0 |
| Upward Shift with Increasing Trend (Type 1) | 0 | 0 | 0 | 0 | 8 |
| Upward Shift with Decreasing Trend (Type2) | 0 | 0 | 0 | 0 | 0 |
| Downward Shift with Increasing Trend (Type3) | 1 | 0 | 0 | 0 | 0 |
| Downward Shift with Decreasing Trend (Type4) | 0 | 0 | 0 | 0 | 0 |

School closure caused no significant change in the level and trend of volume and overtaking in all provinces. Nevertheless, three provinces showed a significant decreasing trend in speeding. The results for tailgating indicated one province with a drop and one with a jump in the level.

The mobility restrictions caused traffic volume to show an increasing trend in many provinces (19 out of 26). The results for tailgating, speeding, and overtaking were mixed; eight out of 26 provinces showed an increasing trend in tailgating while seven provinces experienced a downward shift. In speeding, one province showed an increasing trend while three provinces showed a reduction in the level/trend. In illegal overtaking, two provinces showed a rise and two provinces indicated a decline in the level/trend, respectively.

**Table S2. Results of ITS analysis for speeding in provincial data of Iran**

Intervention

| Observed pattern | Fuel prices | Start of the pandemic | School closure | Imposing restrictions | Removing restrictions |
| --- | --- | --- | --- | --- | --- |
| Only Upward Shift | 0 | 0 | 0 | 0 | 0 |
| Only Downward Shift | 4 | 0 | 0 | 0 | 2 |
| Only Increasing Trend | 0 | 4 | 0 | 1 | 1 |
| Only Decreasing Trend | 3 | 0 | 3 | 2 | 1 |
| Upward Shift with Increasing Trend (Type 1) | 0 | 0 | 0 | 0 | 0 |
| Upward Shift with Decreasing Trend (Type2) | 0 | 0 | 0 | 0 | 0 |
| Downward Shift with Increasing Trend (Type3) | 1 | 0 | 0 | 0 | 0 |
| Downward Shift with Decreasing Trend (Type4) | 4 | 0 | 0 | 1 | 2 |

**Table S3. Results of ITS analysis for tailgating in provincial data of Iran**

| Intervention | Fuel prices | Start of the pandemic | School closure | Imposing restrictions | Removing restrictions |
| --- | --- | --- | --- | --- | --- |
| Only Upward Shift | 0 | 0 | 1 | 0 | 6 |
| Only Downward Shift  Observed pattern | 14 | 0 | 1 | 2 | 0 |
| Only Increasing Trend | 2 | 0 | 0 | 8 | 0 |
| Only Decreasing Trend | 1 | 0 | 0 | 0 | 0 |
| Upward Shift with Increasing Trend (Type 1) | 0 | 0 | 0 | 0 | 7 |
| Upward Shift with Decreasing Trend (Type2) | 0 | 0 | 0 | 0 | 0 |
| Downward Shift with Increasing Trend (Type3) | 1 | 0 | 0 | 5 | 0 |
| Downward Shift with Decreasing Trend (Type4) | 1 | 0 | 0 | 0 | 0 |

Finally, removing movement restrictions caused an increase in either the level or trend of volume in 17 out of 26 provinces. This was observed in half of the provinces for tailgating. Five provinces also showed a reduction in speeding and one province had a significant increase in the trend. Again, the effect of illegal overtaking was mixed, with one province per upward or downward shift/trend.

**Table S4. Results of ITS analysis for illegal overtaking in provincial data of Iran**

Intervention

|  | Fuel prices | Start of the pandemic | School closure | Imposing restrictions | Removing restrictions |
| --- | --- | --- | --- | --- | --- |
| Only Upward Shift  Observed pattern | 2 | 0 | 0 | 1 | 1 |
| Only Downward Shift | 1 | 0 | 0 | 0 | 1 |
| Only Increasing Trend | 3 | 0 | 0 | 1 | 1 |
| Only Decreasing Trend | 3 | 0 | 0 | 1 | 1 |
| Upward Shift with Increasing Trend (Type 1) | 0 | 0 | 0 | 0 | 1 |
| Upward Shift with Decreasing Trend (Type2) | 0 | 0 | 0 | 0 | 0 |
| Downward Shift with Increasing Trend (Type3) | 2 | 0 | 0 | 1 | 1 |
| Downward Shift with Decreasing Trend (Type4) | 1 | 0 | 0 | 0 | 1 |
